# Experiment-based modelling of grain boundary β-phase (Mg_2_Al_3_) evolution during sensitisation of aluminium alloy AA5083

**DOI:** 10.1038/s41598-017-03090-4

**Published:** 2017-06-07

**Authors:** R. Zhang, M. A. Steiner, S. R. Agnew, S. K Kairy, C. H. J. Davies, N. Birbilis

**Affiliations:** 1 Department of Materials Science and Engineering, Monash University, Clayton, VIC 3800 Australia; 2 Materials Genome Institute, Shanghai University, Shanghai, 200072 China; 3 Department of Materials Science and Engineering, University of Virginia, Charlottesville, VA 22904 USA; 4 Department of Mechanical & Materials Engineering, University of Cincinnati, Cincinnati, OH 45221 USA; 5 Department of Mechanical and Aerospace Engineering, Monash University, Clayton, VIC 3800 Australia

## Abstract

An empirical model for the evolution of β-phase (Mg_2_Al_3_) along grain boundaries in aluminium alloy AA5083 (Al-Mg-Mn) during isothermal exposures is proposed herein. Developing a quantitative understanding of grain boundary precipitation is important to interpreting intergranular corrosion and stress corrosion cracking in this alloy system. To date, complete ab initio models for grain boundary precipitation based upon fundamental principles of thermodynamics and kinetics are not available, despite the critical role that such precipitates play in dictating intergranular corrosion phenomena. Empirical models can therefore serve an important role in advancing the understanding of grain boundary precipitation kinetics, which is an approach applicable beyond the present context. High resolution scanning electron microscopy was to quantify the size and distribution of β-phase precipitates on Ga-embrittled intergranular fracture surfaces of AA5083. The results are compared with the degree of sensitisation (DoS) as judged by nitric acid mass loss testing (ASTM-G67-04), and discussed with models for sensitisation in 5xxx series Al-alloys. The work herein allows sensitisation to be quantified from an unambiguous microstructural perspective.

## Introduction

Aluminium (Al) alloy AA5083, based upon the Al-Mg-Mn system, is susceptible to intergranular corrosion (IGC) and intergranular stress corrosion cracking (IGSCC) following prolonged thermal exposure to temperatures between 40 °C and 220 °C^1–5^. Most 5xxx series Al-alloys possess an Mg supersaturation to maximise solid solution strengthening, and the driving force for β-phase (Mg_2_Al_3_) precipitation becomes appreciable when the Mg content is above 3.5 wt.%. As with most Al-alloys in which precipitation occurs, 5xxx alloy precipitates are often associated with (and are of greatest size at) grain boundaries^5–7^, where a lower barrier to nucleation exists. Some details specific to 5xxx series Al-alloys, which merit comment, are that (i) β-phase precipitation offers no age-hardening, instead causing weakening due to solute depletion from the matrix^8^, and (ii) β-phase precipitation also occurs *intra*granularly at heterogeneities including dislocations and Al_*x*_Mn dispersoids (noting that dispersoids are stable and do not respond to thermal exposure)^6, 9^.

When β-phase precipitation occurs at grain boundaries, this electrochemically active phase^10^ will be preferentially dissolved in aqueous environments, contributing to IGC and IGSCC^11–14^; with sensitisation of 5xxx series alloys receiving timely attention^15–18^. It is generally accepted that longer sensitisation times result in more severe IGC damage to 5xxx series alloys on the basis of nitric acid mass lost testing (NAMLT)^19–21^, which is commonly used to assess IGC susceptibility. Most descriptions to date rationalise sensitisation on the basis of temperature and time of exposure, rather than considering quantifiable microstructural parameters, such as grain boundary precipitate size, number density, and area/volume fraction. To fundamentally understand the IGC sensitisation problem, a quantitative understanding of the associated grain boundary microstructure is essential, and microstructural analysis regarding grain boundary β-phase may explicitly determine the relationship between microstructure and IGC damage. Information such as grain boundary β-phase particle radius, number density and nearest neighbour distance, all undergo an evolution as the microstructure proceeds from states which are generically described as ‘discontinuous’ to ‘continuous’. However, the notion of ‘continuous’ remains somewhat contentious since no age-hardenable Al-alloy system offers truly continuous grain boundary coverage by precipitates^5, 12^. The Mg content in alloys such as AA5083 is insufficient to provide enough solute for the formation of a continuous film of finite thickness along the whole grain boundary area (in 3D). An apparently continuous grain boundary β-phase network has often been suggested by 2D sections of polished surfaces (however this represents a very small portion of a complete grain boundary area) or from etched specimens (where the β-phase network is revealed by the degree of grain boundary dissolution, which is greater than the size of the β-phase alone)^22^. A continuous grain boundary network has also been suggested from thin sections viewed in transmission electron microscopy (TEM), where space between precipitates can be obscured by their overlap in 2D transmission^11, 14, 23^. The notion of a fully continuous β-phase network has recently been called into question when observing the grain boundaries in AA5083 via Ga-embrittled intergranular fracture surfaces, where a continuous β-phase is not observed at the grain boundaries.

The modelling of grain boundary precipitates represents a very small portion of the metallurgical field, since most precipitation models are applied to *intra*granular precipitates that provide strengthening. Despite this, several β-phase precipitation models for sensitised AA5083 have been recently reported. The thickness of β-phase along the grain boundary was estimated by Goswami *et al*.^11^, who adopted a Zener-Hillert diffusion controlled growth model of planar interface growth for AA5083. This model was based on the bulk diffusion characteristics of Mg, and therefore, the predicted thickness of β-phase was thinner than that observed by TEM. Notably, the diffusion rate of Mg atoms in the vicinity of grain boundaries could be several orders of magnitude greater than the diffusion rate in the matrix^24^. From the work of Yi and Free, a model^23, 25^ based on the collector plate mechanism and capillarity effects was proposed to predict size and continuity of grain boundary β-phase. In that model, the precipitates keep growing with time until they overlap after 1 month at 70 °C, and this is considered a continuous film. The accuracy of such a model needs to be scrutinised on the basis that complete grain boundary coverage is not empirically observed in any Al-alloy system. Furthermore, in cases where the equilibrium volume fraction of β-phase has been achieved (for a given exposure temperature), phenomena such as ripening require consideration^26–28^. Sensitisation-induced IGC of AA5083 has also been recently modelled based on the Johnson-Mehl-Avarami-Kolmogorov (JMAK) theory^29, 30^. This model relied on an empirical relationship asserting that the DoS is linearly proportional to the β-phase coverage^30^. Such a JMAK model was capable of predicting the relative kinetics of sensitisation (as given by the respective DoS value), however is unable to realistically account for microstructural features (and their variation).

Models developed for sensitisation of AA5083 to date have a premise that at infinite time the boundary condition involves a ‘continuous’ β-phase along grain boundaries. It is important to draw attention to the fact that continuous coverage is not a realistic condition, and while any models with such a boundary condition may offer some utility in the initial stages of sensitisation, in the limiting case they no longer provide any insight into microstructure development. This distinction may seem captious, in regards to initial sensitisation, as all states near full boundary coverage exhibit a similarly high DoS. Despite the claimed observation of continuous β-phase via TEM, there is still no direct evidence for the notion of a continuous film covering all of the grain boundaries^5, 9, 31, 32^. Previous work has revealed that observation of the β-phase using scanning electron microscopy of *inter*granularly fractured 5xxx series alloy surfaces can provide unique information regarding grain boundary β-phase^9, 33, 34^; including a statistical analysis of such grain boundary β-phase. In the present work, we use such empirical data as input for the development of a microstructure-based model for sensitisation of AA5083, with the SEM analysis validated by TEM. A kinetic model was exploited to calculate grain boundary β-phase particle radius, and then modified and iterated, to faithfully represent empirical data collected; providing a robust experiment-based model for sensitisation of AA5083.

## Results

### General microstructure of AA5083 and Electron Back-Scatter Diffraction

**Figure 1:**
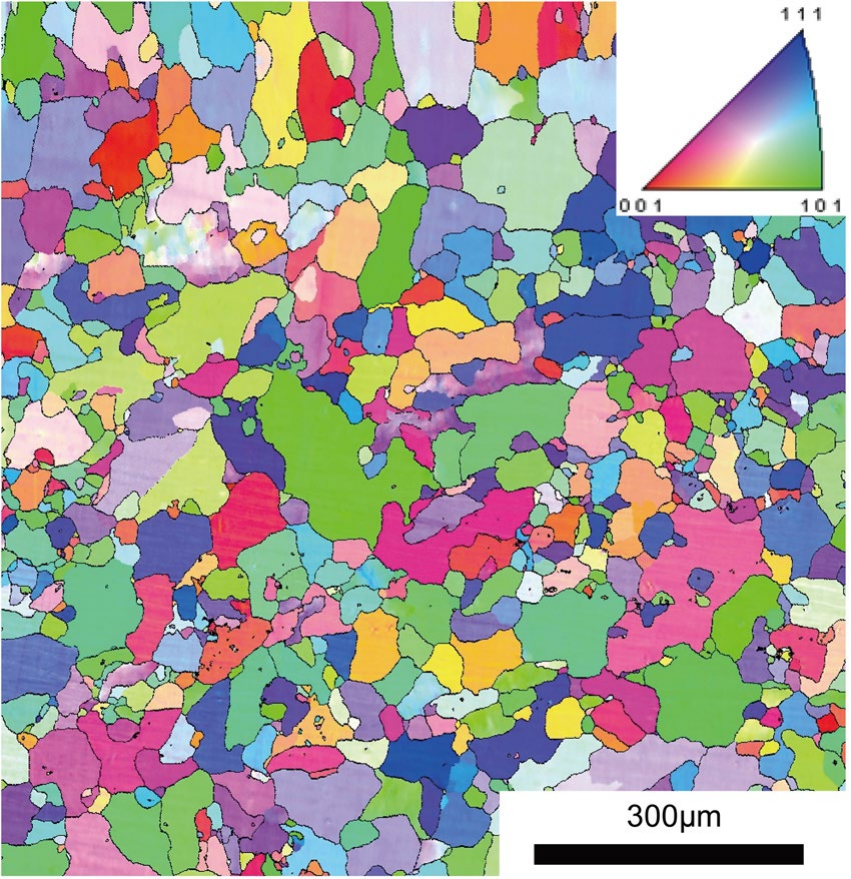
EBSD determined inverse pole figure map for solution treated AA5083 (450 °C/1 hour). The average grain size in the recrystallised microstructure is 63.5 (±22.3) µm.

The typical EBSD derived microstructure and corresponding inverse pole figure for solution heat treated and quenched AA5083 is provided in Fig. 1. The microstructure of the solution heat treated AA5083 is largely recrystallised with an average grain size of ~63.5 (±22.3) µm, and no longer retains the rolling texture. Recrystallisation is evident from the low intragranular orientation spread of recrystallised grains, though a small volume fraction of deformed grains are still present. The subsequently applied isothermal heat treatments (between 80–200 °C) are below the recrystallisation temperature for AA5083, and hence all sensitised samples present share the same grain size distribution and texture as shown in Fig. 1.

### High-Resolution Scanning Electron Microscopy

**Figure 2:**
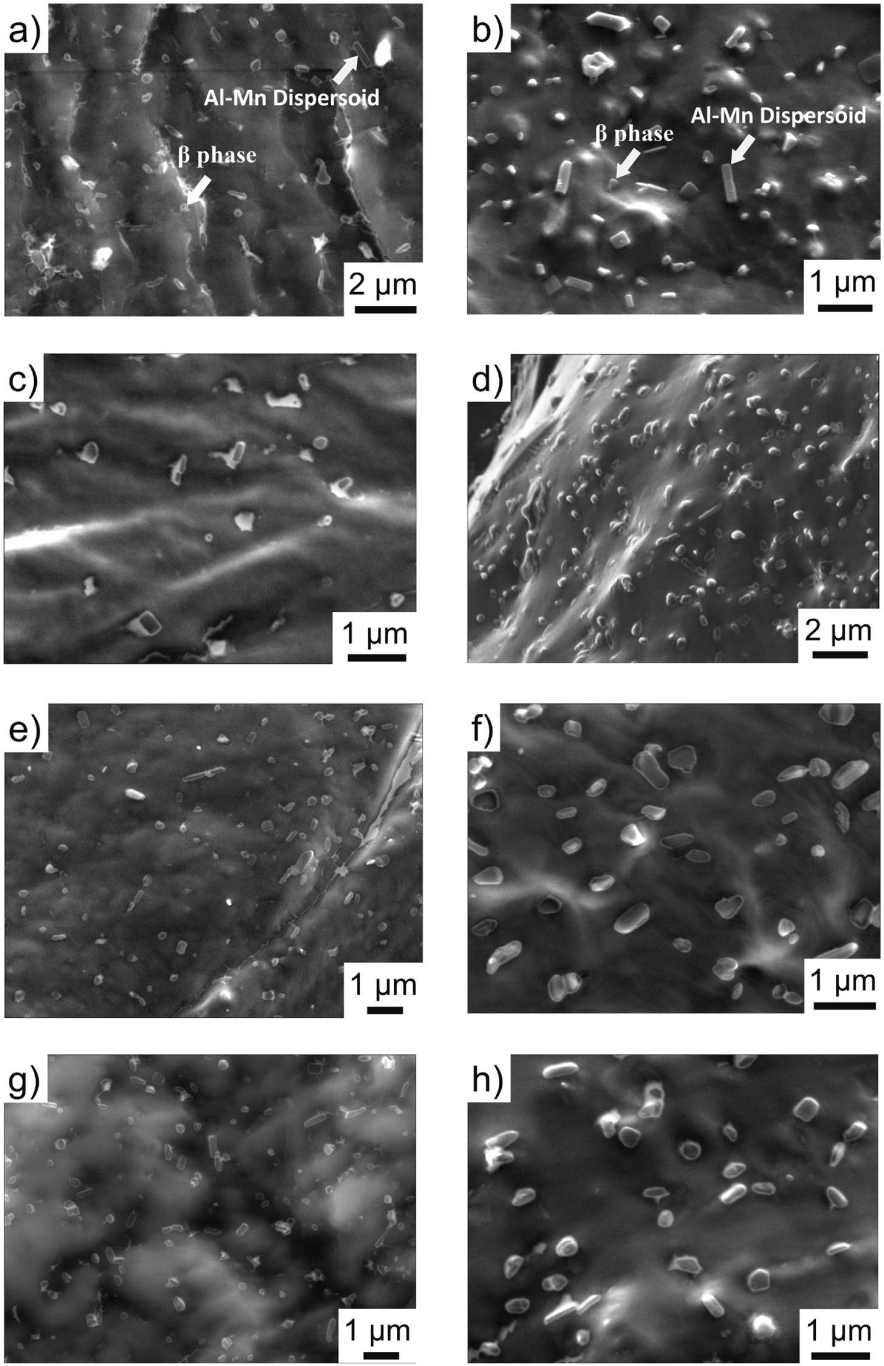
High resolution SEM images of Ga-embrittled fracture surfaces, revealing the grain boundary particles of solution heat-treated AA5083 following thermal exposure: (**a**) 10 days at 80 °C, (**b**) 80 days at 80 °C, (**c**) 2 days at 100 °C, (**d**) 30 days at 100 °C, (**e**) 2 days at 150 °C, (**f**) 8 days at 150 °C, (**g**) 1 day at 200 °C, and (**h**) 10 days at 200 °C. In (**a**) and (**b**) arrows are used to aid the reader in identifying the ‘less regular’ shape of β-phase, as opposed to the Al_*x*_Mn dispersoids which have a regular shape (observed to be square or rectangular) with sharp interfaces. The majority of particles in the images are β-phase.

A set of selected images obtained from Ga-embrittled fracture surfaces is shown in Fig. 2, typical of the various sensitisation conditions that were studied. Arrows have been overlaid on the images to aid in interpretation and to indicate that the majority of particles observed are β-phase, whilst there is also a small fraction of Al_*x*_Mn dispersoids present. The dispersoids are readily detectible due to their unique shape, which is either square or rectangular, with sharp edges. In contrast, β-phase precipitates have a non-unique shape. Further, we also note that the equilibrium volume fraction of the Mn-containing dispersoid phase is very low relative to β-phase (as described and shown in ref. 6) and that the dispersoids are insensitive to thermal exposure in the β-phase sensitisation regime. Upwards of 300 unique β-phase particles were analysed for each sensitisation condition in this study, which is considerably greater than what is typical via TEM analysis. Most of the β-phase precipitates observed reveal an aspect ratio greater than one, and are sub-micron in size. The equivalent particle radius of precipitates is defined as the half the average of the measured longest length. A premise of the present work is that reliable quantitative β-phase information can be ascertained from HR-SEM images of sensitised samples with different thermal histories, from which an empirical model may be established. No continuous β-phase was observed on the Ga-embrittled fracture surfaces, in contrast with previous assertions based upon TEM studies. A salient point is that the imaging herein is pseudo 3D, in that a grain boundary area is studied, as opposed to the cross-sectional view employed in TEM analysis.

**Figure 3:**
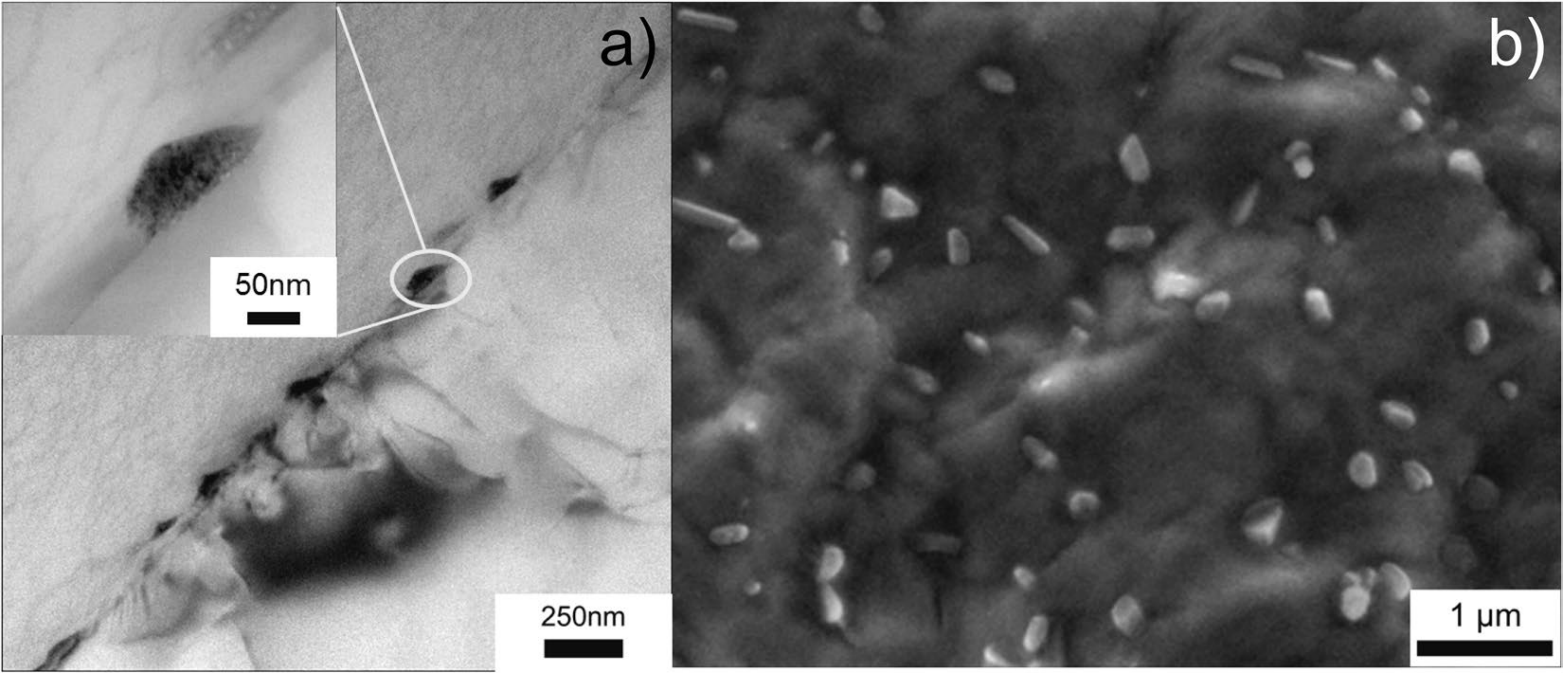
(**a**) TEM image of β-phase precipitates decorating the grain boundary of AA5083 sensitised at 100 °C for 30 days, and (**b**) HR-SEM image revealing the same β-phase from a Ga-embrittled fracture surface of the same specimen from the image in (**a**).

**Figure 4:**
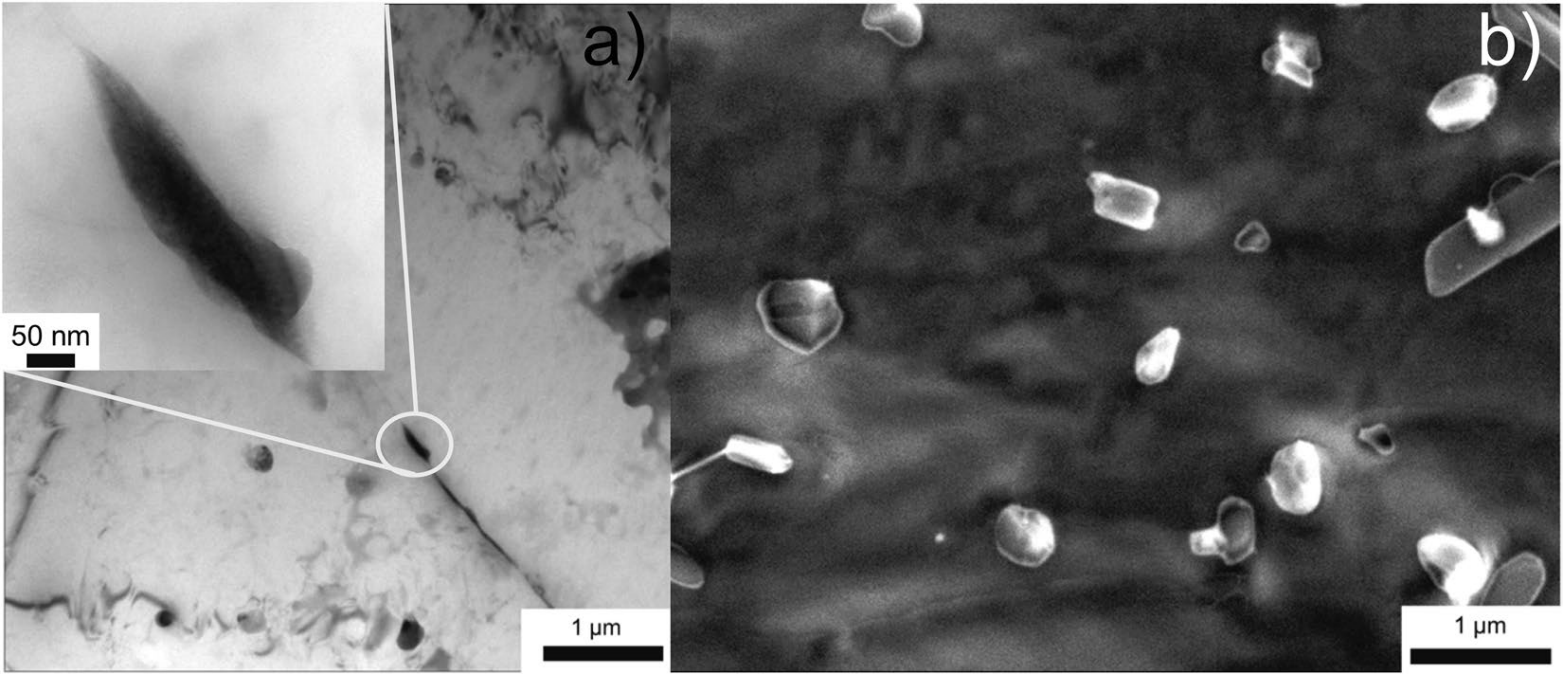
(**a**) TEM image of β-phase precipitates decorating the grain boundary of AA5083 sensitised at 150 °C for 8 days, and (**b**) HR-SEM image revealing the same β-phase from a Ga-embrittled fracture surface of the same specimen from the image in (**a**).

However, in order to benchmark the information acquired from the HR-SEM analysis, a comparison between bright field TEM and SEM images is illustrated in Fig. 3; for a sample heat treated at 100 °C for 30 days with a DoS of around 32.9 (±0.5) mg/cm^2^. The thickness of β-phase in Fig. 3a can be estimated to 50 nm with a length of about ~200 nm; which is consistent with the particles observed in Fig. 3b. In another example, artificial sensitisation was carried out at 150 °C for 8 days, the associated DoS being 50.8 (±0.3) mg/cm^2^, which is higher than that of 100 °C for 30 days treatment. At this relatively severe level of sensitisation, an near continuous β phase decoration of the grain boundary is observed in Fig. 4a, however the HR-SEM image (Fig. 4b) shows that it is not completely continuous.

**Figure 5:**
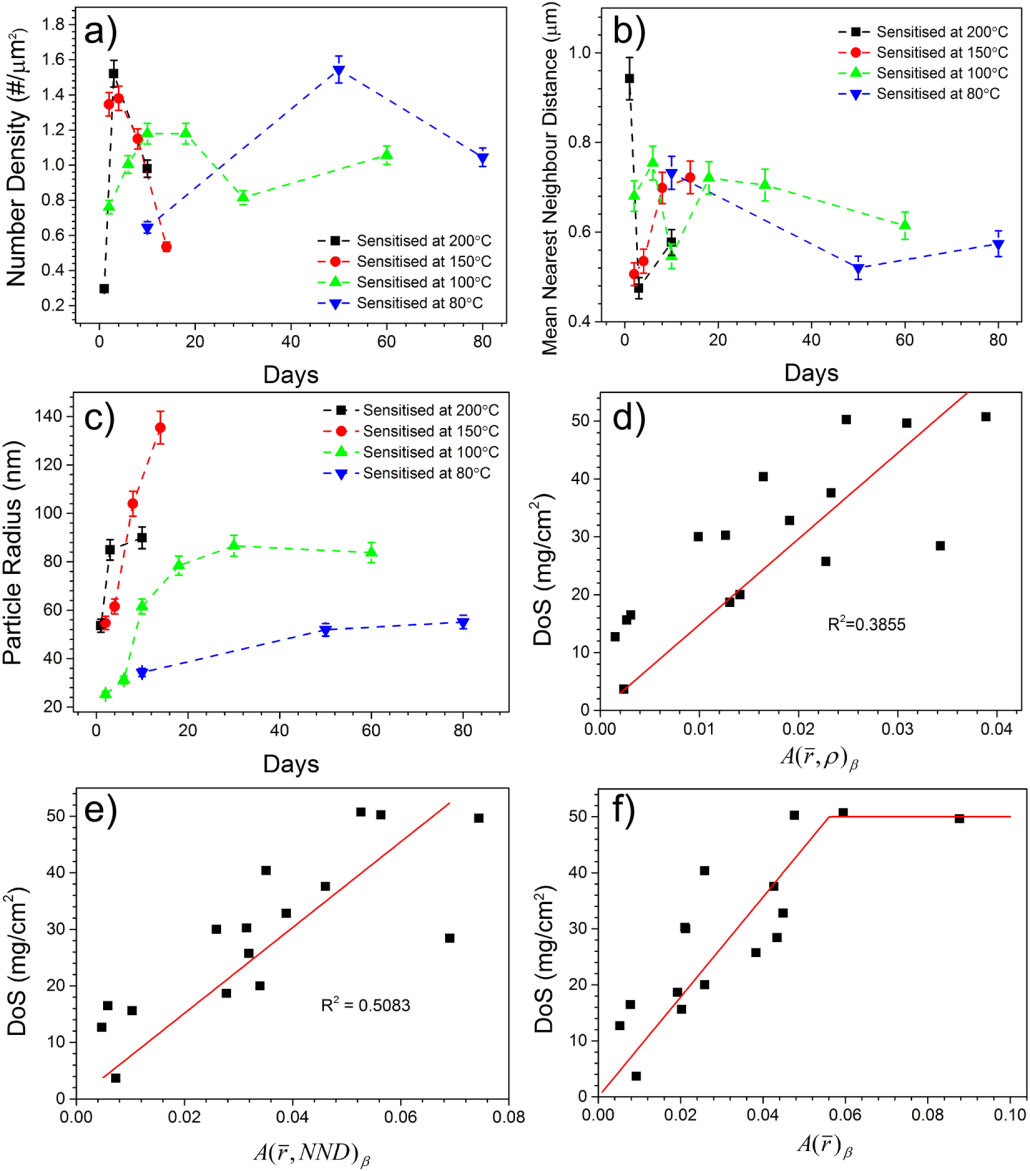
Summary of grain boundary statistics related to β-phase from sensitised AA5083 which was analysed via HR-SEM of Ga-embrittled fracture surfaces. (**a**) Number density of β phase vs. sensitisation time; (**b**) inter-β-phase particle spacing vs. sensitisation time; (**c**) equivalent radius of β vs. sensitisation time; (**d**) DoS (NAMLT values). vs. the areal coverage fraction of β-phase on the grain boundaries $A{(\bar{{\rm{r}}},\rho )}_{\beta }$; (**e**) DoS vs. the areal coverage fraction along the path of greatest particle continuity $A{(\bar{{\rm{r}}},NND)}_{\beta }$; (**f**) DoS vs. the areal coverage fraction assuming a material characteristic NND, $A{(\bar{{\rm{r}}})}_{\beta }$.

A numerical summary for all of the sensitised samples studied is provided in Fig. 5, derived from an extensive experimental matrix to determine a quantitative description of grain boundary β-phase from fracture surfaces imaged by HR-SEM. Based on careful image analysis, Fig. 5a, reveals the number density (ρ) of grain boundary β-phase has a peak value, which appears to occur at later times with lower sensitisation temperatures. It is expected that the number density increases initially due to β-phase nucleation at new sites, and then decreases as precipitates coarsen (at the expense of other, smaller precipitates). Because of the limited data points shown in Fig. 5a, it is not possible to posit a definitive time range for the number density decrease. In addition, by assuming the rugged surface captured by SEM is flat, a certain level of stereological error in the number density is inevitable.

The relationship between the β-phase inter-particle nearest neighbour distance (NND) and sensitisation is not straightforward, as shown in Fig. 5b. This is because the parameter (NND) and the correlated inverse of the particle density ($NND=0.5/\sqrt{{\rm{\rho }}}$ for a random distribution), are affected by any non-random clustering of the particles in the microstructure. As the particles interact through their diffusion fields, they inherently diverge from a perfectly random spatial distribution. In contrast to the complex behaviour of ρ and NND as a function of time and temperature, the SEM data clearly reveals an increase in the average β-phase radius, $(\bar{{\rm{r}}})$, with increasing sensitisation time (Fig. 5c), along with a clear trend in the β-phase radius growth kinetics with increasing temperature.

From these two empirical measurements (number density and average particle radius) it is possible to compute a statistical representation of the areal fraction of β-phase on the grain boundaries. A strong link between β-phase areal coverage and DoS has been previously purported in the literature^9, 35^, and the correlation was recently established using TEM measurements^23^, where continuity/coverage was measured in transmission and approached a “fully continuous” β-phase network at high sensitisation levels. One approach to calculate the areal coverage fraction is to multiply the average particle area, as calculated by the radius, by the density of particles $(A{(\bar{{\rm{r}}},\rho )}_{\beta }=\pi {\bar{{\rm{r}}}}^{2}\cdot \rho )$. This statistic provides a reasonable approximation of the global β-phase coverage fraction, supplying that the distribution of radii is relatively narrow. An alternative statistical areal coverage parameter can be constructed from the NND, ($A{(\bar{{\rm{r}}},NND)}_{\beta }=\,{\bar{{\rm{r}}}}^{2}/{(\bar{{\rm{r}}}+NND/2)}^{2}$), which provides a statistical estimate of the areal fraction of the β-phase along the path of greatest particle continuity. As intergranular corrosion will follow the path of least resistance through the material, the NND adjusted parameter $A{(\bar{{\rm{r}}},NND)}_{\beta }$ (Fig. 5e) correlates marginally better to DoS than the more global parameter $A{(\bar{{\rm{r}}},\rho )}_{\beta }$ (Fig. 5d). As the average NNDs for all of the conditions are relatively similar, with a mean of 644 nm, a third parameter can be constructed from the average radius alone, $(A{(\bar{{\rm{r}}})}_{\beta }=\,{\bar{{\rm{r}}}}^{2}/{(\bar{{\rm{r}}}+644nm/2)}^{2})$, which is the approximate areal fraction of β-phase along the path of greatest particle continuity assuming that the particles form with a characteristic spacing (Fig. 5f). It is worth noting that the assumption of a constant average NND in AA5083 is consistent with site-saturated nucleation of the β-phase, which has been proposed previously based on JMAK modelling^29, 30^. DoS values rise steadily with the $A{(\bar{{\rm{r}}})}_{\beta }$ parameter until reaching a plateau of about 50 mg/cm^2^, as is commonly observed in other data sets, and can be represented by the equation:

1 $$\begin{array}{c}DoS(mg/c{m}^{2})=892\,A{(\bar{{\rm{r}}})}_{\beta }\,when\,A{(\bar{{\rm{r}}})}_{\beta }\le 0.056\\ DoS(mg/c{m}^{2})=50\,when\,A{(\bar{{\rm{r}}})}_{\beta } > 0.056\end{array}$$

Note that the areal coverage fractions of all these parameters are less than 10% for even the highest DoS values, in contrast to previous conceptions of β-phase forming a continuous network. This realisation is critical in evolving towards an unambiguous metallurgical model for grain boundary precipitation that is faithful to the physical observation of grain boundaries and the discrete nature of precipitates.

### Sensitisation Modelling

#### β-phase precipitation model

**Figure 6:**
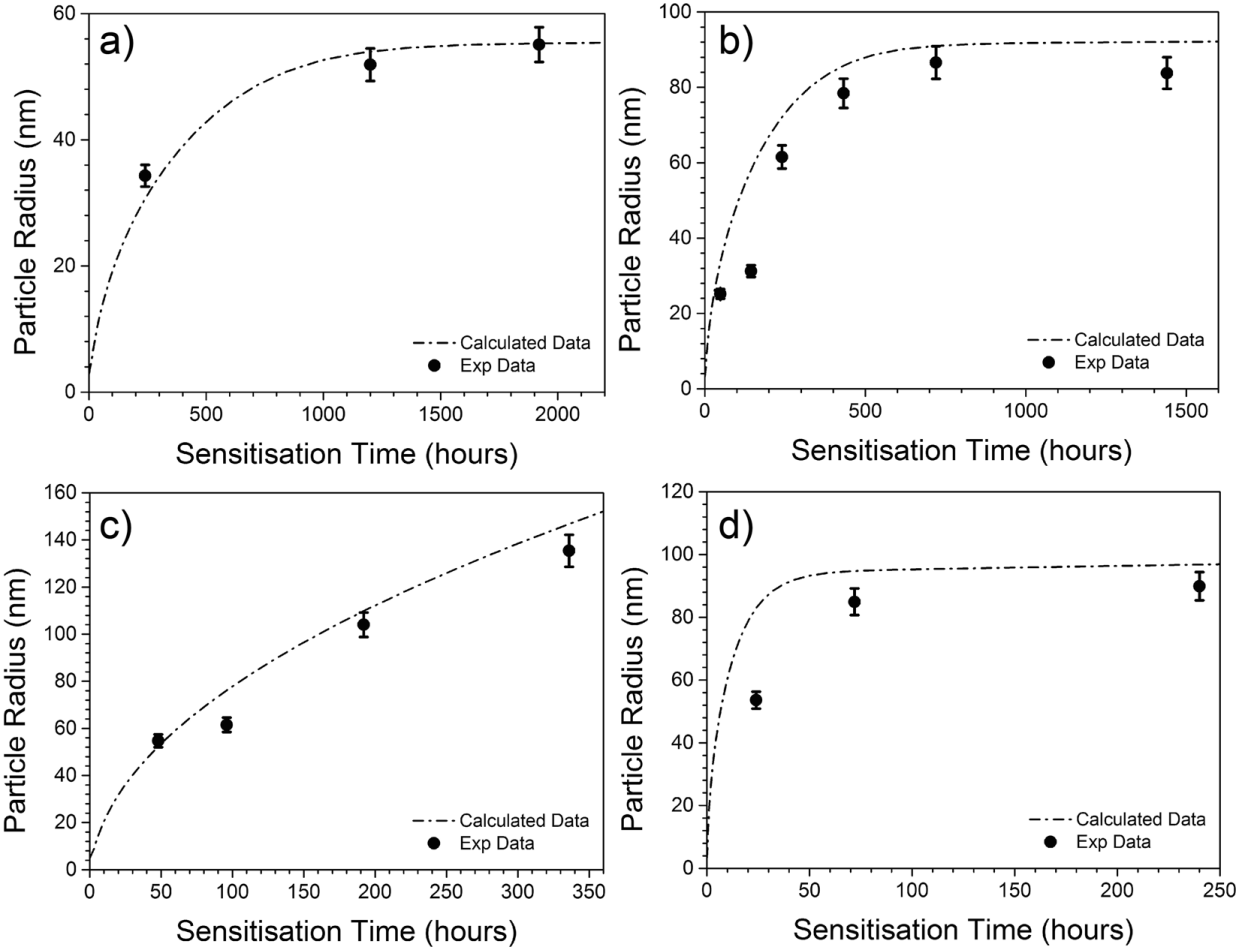
Average radius of grain boundary β-phase (Mg_2_Al_3_) from HR-SEM images and simulation of particle radius from a modified CALPHAD based model for AA5083 sensitised at (**a**) 80 °C (**b**) 100 °C (**c**) 150 °C and (**d**) 200 °C for various times. The standard deviation of the mean particle radius is between 20% and 35%.

A CALPHAD-based model tuned to the microstructural information collected from grain boundary β-phase precipitate analysis above is presented in Fig. 6. The model was constructed to predict the $(\bar{{\rm{r}}})$ parameter, which typifies the grain boundary β-phase state. The construction of such a model was executed using the PanPrecipitation model that employs the PANDAT database to provide equilibrium thermodynamic data, such as the maximum volume fraction of β-phase possible at a given temperature. It merits comment that such precipitation models which employ thermodynamic data and follow the classical nucleation and growth principals were originally used (and validated) for *intra*granular precipitation modelling in Al-alloys, as previously reported by Grosvenor^36^. As such, we seek to use a modified version of the classical model in order to fit the empirical grain boundary precipitate data.

A detailed description of the CALPHAD-based precipitation model being utilised can be found in ref. 37. The homogeneous nucleation rate (*J*) for can be described by classical nucleation theory as:

2 $$J={N}_{v}Z{\beta }^{\ast }\exp (-\frac{{\rm{\Delta }}{G}^{\ast }}{kT})\exp (\frac{-\tau }{t})$$

Where *N*_*v*_ is the nucleation site density, Z is the Zeldovich factor, *β* ^*^ is the atomic attachment rate, *k* is the Boltzmann constant, *T* is the absolute temperature (in K), *τ* is the incubation time for nucleation and *t* is time. In addition, Δ*G* ^*^ is the critical nucleation energy for homogeneous condition which can be expressed as:

3 $${\rm{Driving}}\,{\rm{force}}\,{\rm{parameter}}\cdot \frac{4\pi }{3}{({r}^{\ast })}^{2}\sigma $$

where *r* ^*^ is the critical precipitate radius and *σ* is the interfacial energy of the matrix- precipitate interface. For heterogeneous nucleation, which is relevant to grain boundaries, both the shape factor which will affect the activation energy barrier for heterogeneous nucleation and the increased nucleation site density should also be considered. As a first approximation, the above two aspects can be accounted for by employing Δ*G* ^*^ in Eq. (2), as a fitting parameter, termed the ‘driving force parameter’.

The growth rate of precipitate particles can be determined according to:

4 $$\frac{dr}{dt}=\,\frac{D}{r}\,\frac{{c}_{i}-{c}_{r}^{\alpha }}{{c}^{\beta }-{c}_{r}^{\alpha }}$$

In Eq. (4), *r* is the precipitate radius, *D* is the diffusivity of Mg in the matrix, *c*_*i*_ is the instantaneous concentration of solute in the matrix, ${c}_{r}^{\alpha }$ is the concentration of solute in the matrix at the precipitate interface, whilst *c* ^*β*^ is the concentration of solute in the precipitate. By considering the Gibbs-Thomson size effect, the growth rate can be further modified as:

5 $$\frac{dr}{dt}=\frac{K}{r}(\frac{1}{{r}^{\ast }}-\frac{1}{r})$$

and a so-called kinetic parameter *K* can be expressed as:

6 $$K=\frac{2\sigma {V}_{m}}{({\rm{\Delta }}{C}^{\alpha \beta }]{[M]}^{-1}({\rm{\Delta }}{C}^{\alpha \beta }]}$$

The parameter *V*_*m*_ represents the molar volume of the precipitate phase, the row vector $({\rm{\Delta }}{C}^{\alpha \beta }]$ and column vector $({\rm{\Delta }}{C}^{\alpha \beta }]$ separately represent the concentration difference of *α* and *β*, and [*M*] is the chemical mobility matrix^38^.

Again recalling that the situation for *inter*granular precipitates is unique, as the above is expressions were established for the case of *intra*granular precipitates - the following modification for expressing the rate of grain boundary precipitation was needed:

7 $$\frac{dr}{dt}=\frac{D}{r}\,\frac{{c}_{i}-{c}_{r}^{\alpha }}{{c}^{\beta }-{c}_{r}^{\alpha }}\,\frac{{D}_{gb}\sqrt{t}\sqrt{D}}{{\pi }^{2/3}{r}^{2}}\,$$

where *D*_*gb*_ is the grain boundary diffusion coefficient. Whilst the diffusion rate of Mg in Al is reported in the literature, we note that the grain boundary diffusion rate of Mg in Al is not reported, and as a result, the model as described herein was iteratively “tuned” with experimental data. Three fitting parameters were employed; namely the kinetic factor, diffusion factor and driving force factor; which are given by *K*, *D* and the aforementioned ${\rm{\Delta }}{G}^{\ast }$.

The correlations between the CALPHAD-based model and the experimentally measured values for four different sensitisation temperatures (80 °C, 100 °C, 150 °C and 200 °C) are shown in Fig. 6. The form of the modelled data provides insightful aspects for grain boundary β-phase precipitation. For example, when used to model the time evolution of the average particle radius $(\bar{{\rm{r}}})$ it appears that the β-phase growth rate will decrease after prolonged sensitisation at 80 °C, 100 °C and 200 °C, whilst β-phase appears to sustain an appreciable growth rate at 150 °C, even after 14 days sensitisation. Comparison of the experimental data and the model regression presented in Fig. 6 show that the form of the CALPHAD-based model is satisfactory.

**Figure 7:**
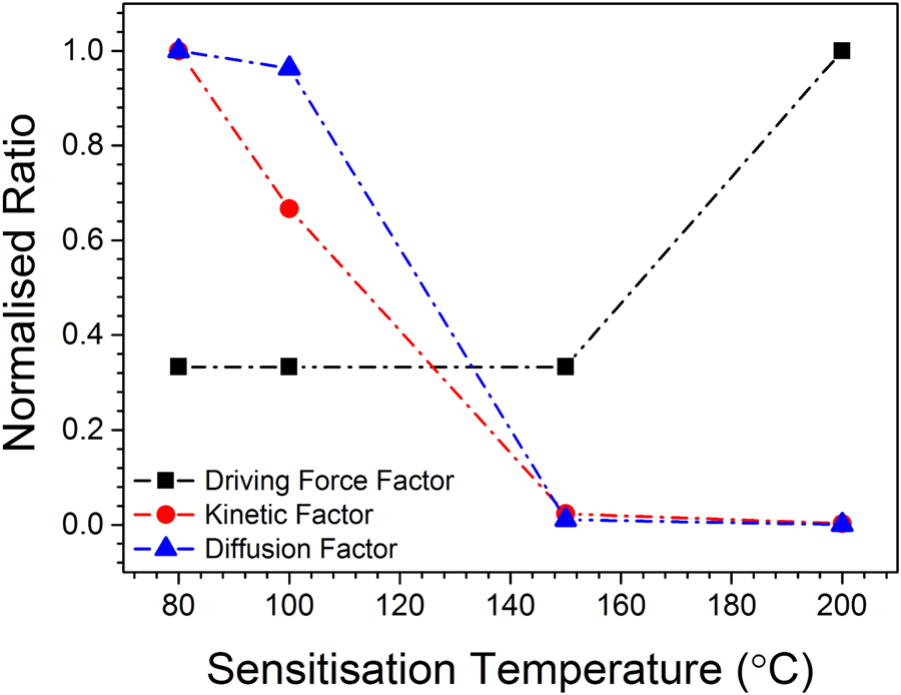
Empirical factors imposed on the CALPHAD model to provide a fit to normalised driving force factor, kinetic factor and diffusion factor applied to modify the CALPHAD model to faithfully represent the experimental data and subsequently provide an analytical model to represent the specific case of grain boundary precipitation.

The variation in the free variables *K*, *D* and ${\rm{\Delta }}{G}^{\ast }$ is presented in Fig. 7. The variations in such parameters with time are rationalised on the basis that they are inter-related according to: (i) the equilibrium volume fraction for β-phase formation is dramatically decreased as the temperature increases from 80 °C to 200 °C, (ii) the relative diffusion factor is altered with sensitisation temperature as the relative solubility of Mg in the matrix varies, and (iii) with increasing temperature, particle nucleation becomes more difficult (where the driving force has a direct proportionality to interfacial energy).

**Figure 8:**
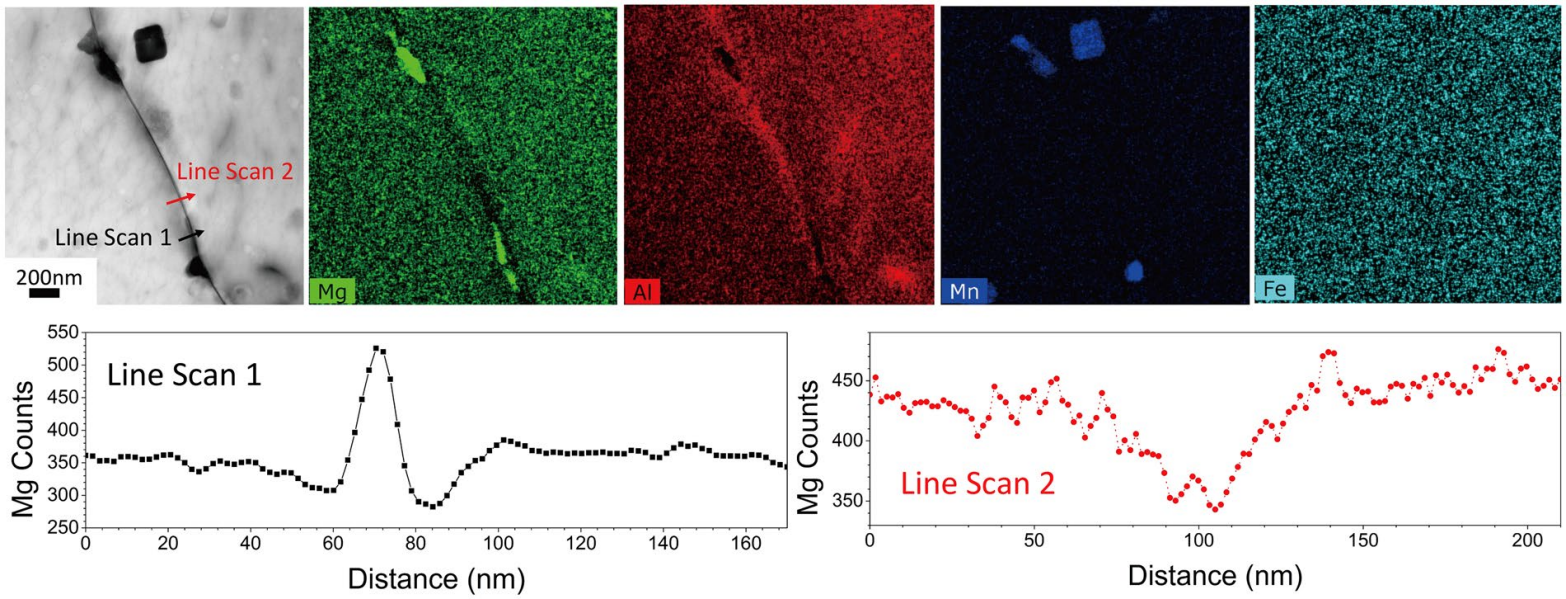
Scanning TEM image showing grain boundary decorated with β-phase precipitates in AA5083 sensitised at 100 °C for 30 days; along with the corresponding EDS map of Mg, Al, Mn and Fe. Line profiles are also presented for the variation in Mg for the lines denoted in the TEM image. Line Scan 1 shows the increase in Mg content across a β-phase precipitate, whilst Line Scan 2 reveals the Mg depletion across the grain boundary in a region with no β-phase precipitates.

**Figure 9:**
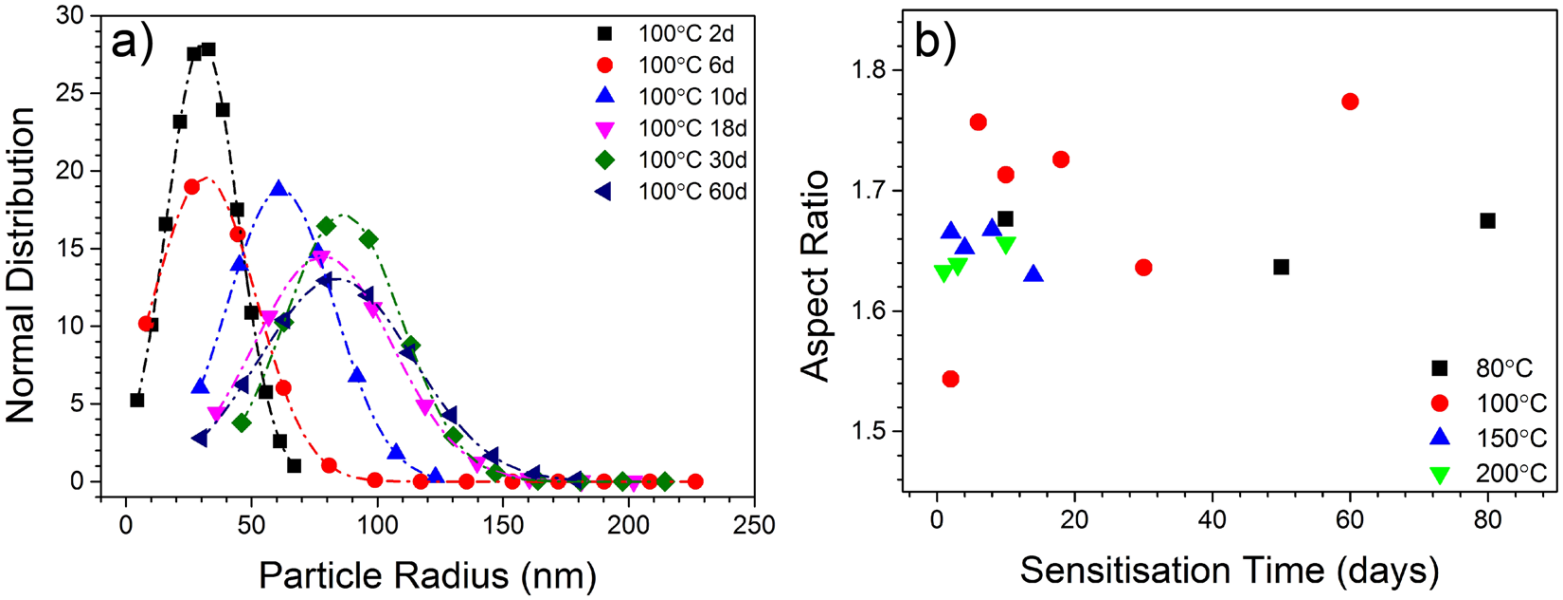
(**a**) The normal distribution of grain boundary β-phase particle radius in AA5083 which sensitised at 100 °C for 2 to 60 days, (**b**) the average aspect ratio of β-phase particles observed on Ga-embrittled fracture surface of alloys with different sensitisation heat treatments.

In order to rationalise the data herein, as illuminated from the CALPHAD modelling, a number of further tests were carried out to provide somewhat more physical consideration of model predictions and use of free variables. It can be observed that the kinetics of β-phase growth are reduced with sensitisation time (i.e. the relative rate of change of $\bar{{\rm{r}}}\,\,$is more rapid at early sensitisation times), shown in Fig. 6. To rationalise this, Scanning TEM and EDS line profiles and mapping were carried out upon a sensitised AA5083 specimen (Fig. 8). It was validated that there is a depletion of Mg in the region of grain boundaries where no β-phase exists, and that there is a slight (but determinable) Mg depletion immediately adjacent to grain boundary β-phase precipitates (the precipitates themselves having Mg enrichment as expected). Such an Mg profile confirms that β-phase growth is limited by the bulk diffusion of Mg to the boundary, the rate of which will slow with time (∝ t^1/2^). The size of the depletion zone in Fig. 6 is consistent with estimates of the bulk diffusion of Mg in FCC-Al^39^, which predict a mean diffusion length of 75 nm on either side of the grain boundary for this sample. Grain boundary Mg depletion (with the exception of where ripened β-phase exists) will also supress the nucleation of any new β-phase precipitates. To this end, we observed that the grain boundary β-phase number density decreased after crossing a maximum (Fig. 5a). The decrease in number density was attributed to the dissolution of smaller β-phase precipitates (those below a critical radius r*) by the growth of β-phase precipitates which possess a critical radius > r*. The normal distribution of grain boundary β-phase size is given in Fig. 9a, where the mean radius is shown to increase, and the distribution tends to indicate the dissolution of what were originally smaller β-phase particles. The evolution of the average aspect ratio (of longest diameter to shortest diameter) of grain boundary β-phase particles is also presented in Fig. 9b. The β-phase shape, whilst varying within a finite range, is generally confined to an aspect ratio of ~1.65. Such microstructural and microchemical factors, and their study, merit further work in order to rationalise the use of the free variables employed herein to give weightings to the classical precipitation model as applied to grain boundaries.

On the basis that the presented CALPHAD-based model is a faithful representation of the grain boundary β-phase growth kinetics as a function of sensitisation temperature, an empirical model that contains only sensitisation temperature and sensitisation time as input parameters can be proposed. This empirical model simply combines the results of the CALPHAD modelling tuned for grain boundary β-phase executed at unique temperatures. The equation for the empirical model may be given as:

8 $$\bar{{\rm{r}}}=-0.7261T(^\circ C)\cdot \exp (\frac{t(hr)}{-0.7986T(^\circ C)})+1.011T(^\circ C)-27.63$$

**Figure 10:**
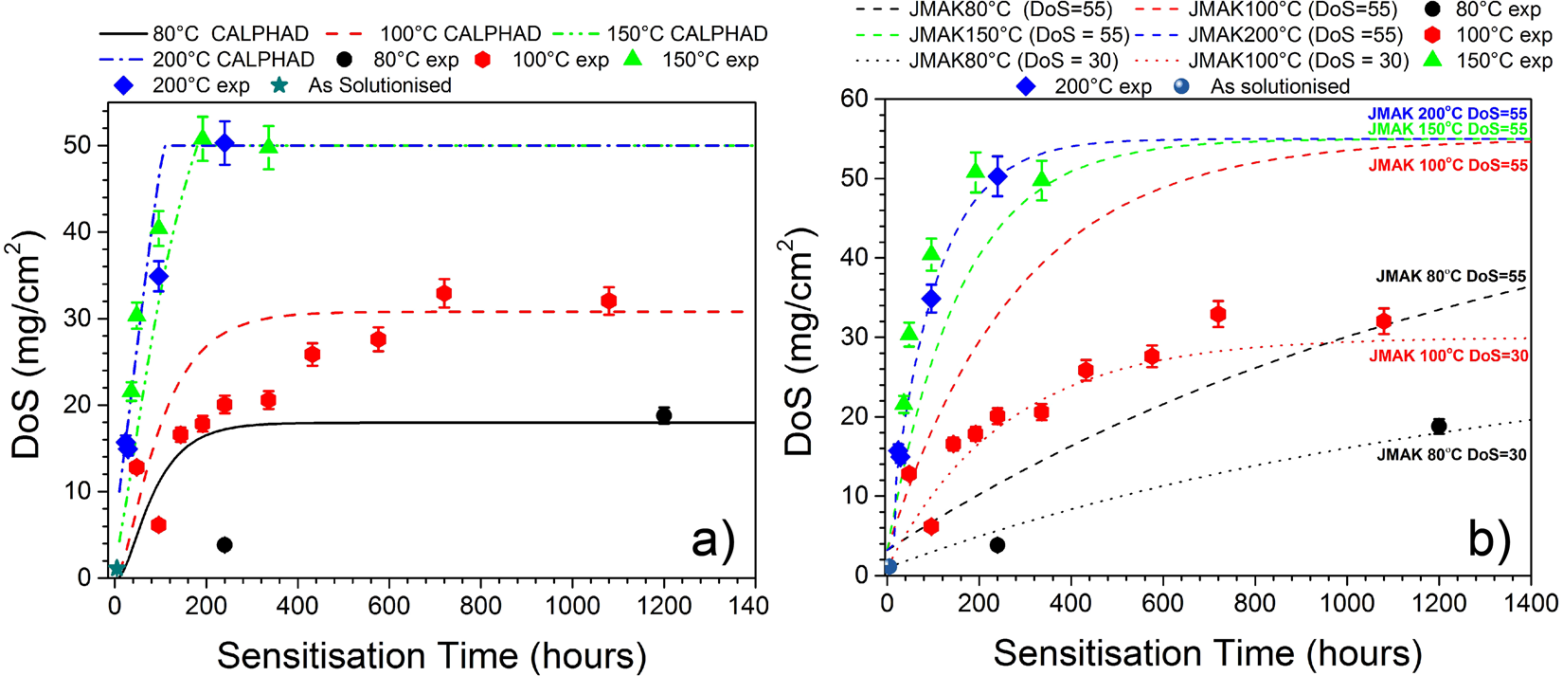
(**a**) The lines represent DoS values predicted by the CALPHAD model which is yielded by Eqs (1) and (8), and the experimental DoS values are presented in the form of scatter data points. (**b**) Degree of sensitisation (NAMLT values) as a function of isothermal aging time for AA5083. The dotted line for JMAK 40 °C ~ 100 °C represents estimated DoS of sensitised AA5083-H116 from a the JMAK model presented in ref. 29, whilst the JMAK 150 °C and 200 °C lines are calculated according to the values in Fig. 11.

In this equation, T represents sensitisation temperature in degrees Celsius, t is the sensitisation time in hours, and the output is the average grain boundary β-phase particle radius $(\bar{{\rm{r}}})$ in nanometres. Having previously established a relationship between $\bar{{\rm{r}}}$ and DoS in Eq. (2), it is now possible to make sensitisation predictions from the CALPHAD model and compare them to experimental data in Fig. 10a. It can be found that the calculated DoS can accurately predict the experimental data at elevated sensitisation temperature (≥150 °C), however the model is less sensitive to the low temperature sensitisation, slightly over-predicting the resultant DoS at relatively short sensitisation times. This distinct response with low sensitisation temperature may be caused by the confluence of decreased Mg diffusion kinetics at lower temperature, coupled with a higher equilibrium β-phase fraction; the latter causing an increase in the possible number of β-phase nucleation sites.

#### JMAK model

**Figure 11:**
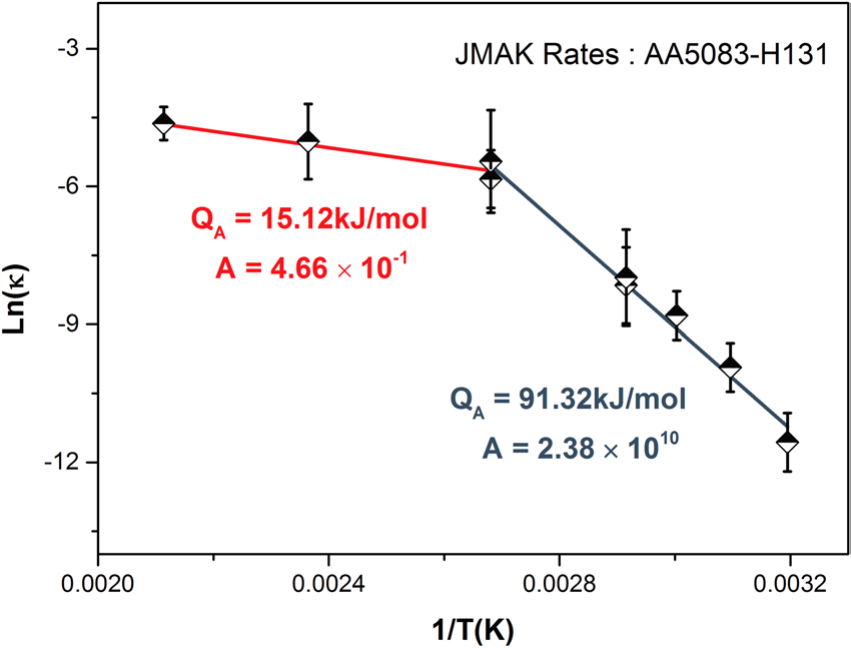
Arrhenius plot of the rate constants for AA5083 which were sensitised between 80 and 200 °C. A change in the kinetics of sensitisation was observed.

The experimental data presented in herein can also be analysed by the recently presented JMAK based model for sensitisation^28, 29^, providing some juxtaposition to the CALPHAD-based results. The JMAK model was originally developed explicitly for low temperature sensitisation, as it was noted from the limited high temperature data available that the kinetics of sensitisation seemed to experience a change above 100 °C ^26^. Coincidently, the DoS measurements in this study provide the necessary data to establish high temperature kinetics for AA5083-H131 and expand the JMAK model. The JMAK model predicts the sensitisation process through the fundamental relations:

9 $$X(t)={(\kappa (T)t)}^{n}$$

10 $${\rm{\kappa }}({\rm{T}})=A\,\exp (-{{\rm{Q}}}_{{\rm{A}}}/{{\rm{k}}}_{{\rm{B}}}{\rm{T}})$$

where X is the fraction of the grain boundary coverage, *κ*(*T*) is a temperature dependent rate constant, *Q*_*A*_ is a combined effective activation energy encompassing both nucleation and growth contributions, *A* is a constant that incorporates a number of factors including precipitate shape, nucleation rate, and diffusion, and *n* is the JMAK time exponent. The JMAK model has a fixed n = 1 for the AA5083-H131 temper, and the remaining two rate constants, *Q*_*A*_ and A, can be fitted from the Arrhenius relationship in Eq. 10. Adding the 150 °C and 200 °C DoS series to prior data provided in ref. 26, it is clear that AA5083-H131 experiences a marked shift in kinetics above 100 °C (Fig. 11). The change in kinetics at higher temperatures could arise from a number of different sources; including the decrease in the driving force present to nucleate the β-phase, and the increased likelihood of Mg content forming *intra*granular precipitates ^26^.

The underlying basis of the JMAK model is grounded in the impingement of growing precipitates, which in the model’s original formulation eventually form a continuous network along the grain boundaries ^28, 29^. In consideration of this study’s findings, demonstrating that a continuous network of the β-phase does not exist, it is important to re-approach the prior physical interpretation of the JMAK model. Rather than the precipitates physically impinging upon each other, it is instead their local environments (where they sensitise the grain boundaries) that can begin to overlap until the whole boundary is sensitised in a soft impingement process. As all the parameters of the JMAK model are empirically determined, this change has little consequence to the model outside of the physical interpretation of the fitted parameters.

Converting the grain boundary coverage estimates provided by the JMAK model to DoS values produced by NAMLT test requires introducing a set of scaling parameters. This is complicated by the large degree of variation observed in the literature between data series reported for hypothetically identical materials experiencing the same isothermal holds. Such variation may be caused by a number of factors, ranging from nitric bath temperatures and exfoliation procedures, to unaccounted microstructural differences between material lots^30^. The uncertainty provided by these variables makes modelling of raw sensitisation values from first principles, using either the JMAK or CALPHAD-based model, exceedingly difficult. In the case of the 100 °C experimental DoS values measured in this study and presented in Fig. 10, the sensitisation of the AA5083-H131 material plateaus at 30 mg/cm^2^. Two previously published AA5083-H131 100 °C DoS series in the literature exhibit a contradictory plateau of approximately 55 mg/cm^2^ ^21, 40^. For these reasons, it was found that the JMAK model was very accurate at predicting the relative kinetics of sensitisation $(\overline{{{\rm{R}}}^{2}}=0.965)$ but would often be a poor fit for individual series if they exhibited a considerable deviation from the approximate average scaling factors (DoS = 3.2 mg/cm^2^ and DoS = 55 mg/cm^2^ for zero and full coverage of AA5083-H131, respectively) as seen for the 100 °C DoS series in Fig. 8 ^30^. Allowing the scaling parameters to be set for a particular data series, for example DoS = 1 mg/cm^2^ and DoS = 30 mg/cm^2^ as observed for the 100 °C DoS series in Fig. 10b), demonstrates that the JMAK model is generally able to reproduce the functional form of sensitisation even if it is a poor predictor of the raw DoS values of some series.

## Discussion

Herein we have presented two experimentally informed models for assessing the time and temperature dependant grain boundary sensitisation of AA5083. It is emphasised that no continuous grain boundary β-phases was observed for sensitised samples examined in this work with DoS ranging from 3.2 mg/cm^2^ to 51.1 mg/cm^2^ (i.e. Fig. 2), contrary to assertions in much of the previous literature. Instead, HR-SEM images reveal physically distinct β-particles distributed with a high number density. For example, specimen sensitised at 100 °C for 30 days show discrete β-phase precipitates ~50 nm in thickness and ~150 nm in length, (both in SEM and TEM observation, Fig. 3). In light of this finding, many conclusions regarding the geometry of β-phase precipitates on 5xxx series grain boundaries may need to be revisited in future works.

There is no obvious relationship for either the β-phase number density or average nearest neighbour distance (NND) with time (Fig. 5a and b). However, the β-particle size (as judged by the equivalent radius) in Fig. 5c increases as sensitisation time increases.

A statistical parameter representing the areal fraction of β-phase along the path of greatest particle continuity can be established from the β-phase particle radii, and is seen to correlate with the DoS (from ASTM-G67-04) in Fig. 5f. This is considered an important finding, as time and temperature independent representations of DoS that rely on an experimentally confirmed microstructural state have been absent in the literature. In the present work we have not elaborated aspects such as any transition to SCC and the role of practical exposure environment. How SCC cracking propagates in 5xxx series Al-alloys presently remains under debate, with certain works indicating the contribution of hydrogen embrittlement in combination with anodic dissolution of β-phase^41^.

There is a considerable amount of information presented in Fig. 10, where both the CALPHAD and JMAK models for sensitisation are compared to experimentally measured DoS values. The CALPHAD-based precipitation model was shown to predict the overall degree of sensitisation at 100 °C, 150 °C and 200 °C, but with slightly less fidelity at 80 °C. One possible explanation is that IGC is not significantly affected by the particle size below a certain threshold. It is also worth noting that the equilibrium volume fraction of β-phase at low temperatures (such as at 80 °C) is high – ~10 vol. % according to the CALPHAD model employed – however it is likely that the equilibrium volume fraction of β-phase is not approached within the time frame of the tests performed at 80 °C, and this factor would likely contribute to increased rather than decreased IGC in the sample.

A JMAK model, with the high temperature expansion presented, was able to predict the overall kinetics of AA5083-H131 sensitisation, though it remains unable to account for variations in the sensitisation plateau values observed between all reported studies (e.g. sensitisation studies 100 °C) using only literature averaged scaling factors. The original derivation of the JMAK model for predicting sensitisation was based upon physical impingement of precipitates. This view can now be revised for the scenario of a discrete, non-continuous network of precipitates as observed in this study. If each β-phase particle on a grain boundary can be considered to have a finite area of influence which it sensitises, instead of modelling the impinging growth of precipitates, the JMAK model can instead be considered to model the impinging regions of precipitate influence. In a similar vein to the empirically derived continuity parameter presented herein, this would replace the concept of full boundary coverage by a continuous film of the β-phase, with the boundary being fully covered by the network of discrete precipitates and their overlapping regions of influence. Indeed, this shift in understanding only alters the interpretation of the fitted rate constants, *Q*_*A*_ and A, rather than the model itself.

Comparing the classical nucleation CALPHAD-based model and the JMAK model, which relies on a microstructurally determined seeding of heterogeneous nucleation sites, the ability of the JMAK model to faithfully represent the sensitisation kinetics suggests that sensitisation proceeds by heterogeneous site saturated nucleation as opposed to the classical homogeneous nucleation theory. Nonetheless, consideration of both models is useful to provide a detailed consideration of grain boundary precipitation phenomena, and the challenges in modelling grain boundary precipitation phenomena, which herein could only be achieved by having experimental benchmarks. It may be possible in the future to integrate the concept of soft impingement of the regions of influence into a refined fundamental physics-based model of precipitate size, which builds upon the lessons learned in the present empirical study.

We emphasise that the proposed experiment based model herein is only aimed for AA5083 with a recrystallised grain structure. In a general sense, the texture^1, 42^, grain size, and level of cold work of specimens will also influence the DoS for sensitised AA5083, however such parameters are not presently discussed and will be considered in future iterations for the modelling of grain boundary precipitation of β-phase. However, the model framework herein can be useful for comparing cases where the bulk composition of the alloy is modified, in cases where one seeks to determine the value of remedial (reversion) heat treatments, and to account for aspects such as grain boundary length or area (which vary with grain size) and texture. The linear relationship between the grain boundary β-phase coverage and the propensity for IGC in Al-alloys merits further focused studies.

## Conclusions

- High-resolution SEM analysis of grain boundaries was carried out following preparation of intergranular surfaces, using a Ga-embrittlement technique on a series of samples subjected to a test matrix of isothermal aging times. The approach was able to provide statistically relevant characterisation of grain boundary β-phase, which was also benchmarked from TEM examination. This paper provides one of the most comprehensive empirical reports of 5xxx grain boundary β-phase precipitate statistics to date.
- No continuous β-phase ‘film’ was observed for sensitised samples examined in this work with the specimens studied presenting DoS values ranging from 1.2 mg/cm^2^ to 51.1 mg/cm^2^.
- The empirical grain boundary β-phase information collected allowed for the development of a thermodynamic, CALPHAD-based model for sensitisation in AA5083 that was fitted to experimentally verified microstructural features. This model can be used not only to predict the DoS (as determined from NAMLT testing), but also the radius of β-phase precipitates at the grain boundary as a function of time and temperature.
- The empirical results in this study were also used to refine and expand upon a previously proposed DoS model based upon the JMAK theory, adding the ability to predict sensitisation at temperatures above 100 °C and refining the physical interpretation of parameters in the original model.
- Comparison of the CALPHAD and JMAK models suggests that grain boundary sensitisation proceeds by heterogeneous, site-saturated nucleation.

## Methods

### Materials

AA5083-H131 thick plate was investigated in this study, the composition of which was independently measured using inductively coupled plasma atomic emission spectroscopy (ICP-AES, Spectrometer Services, Coburg, VIC, Australia) and listed in Table 1. Samples were cut from the center of the plate (T/2 position), solution treated in a salt bath at 450 °C for 1 hour (to eliminate any existing β-phase), and then water quenched. Such specimens were designated as ‘solution treated’. To sensitise the specimens, isothermal heat treatments were applied to the solution treated and quenched material at 80 °C, 100 °C, 150 °C and 200 °C for various durations (up to 80 days). The degree of sensitisation (DoS) following heat treatment was evaluated by Nitric Acid Mass Loss Testing as per ASTM-G67-04.

**Table 1:** Composition of AA5083 used in this study (determined by ICP-AES).

	Mg	Mn	Fe	Si	Cr	Cu	Zn	Ti	Al
wt. %	4.32	0.6	0.21	0.1	0.08	0.05	0.03	0.02	Bal.

### Characterisation

Scanning electron microscopy and electron backscatter diffraction (EBSD) were conducted using an FEI Quanta 3D FEG, equipped with a Pegasus Hikari EBSD system. The morphology and size of grains was analysed using TSL^®^ orientation image mapping software. Specimens for EBSD analysis were prepared metallographically to a 0.05 µm finish, followed by a final surface preparation by ion milling using a GATAN precision etching coating system (PECS^TM^). Prior to the characterisation of grain boundary β-phase, specimens were cut into a rectangular samples of 53 mm × 8 mm × 8 mm, and then *inter*granularly fractured following a Ga-embrittlement treatment^5, 9^. This procedure yields an area of *inter*granular fracture and allows for statistically relevant populations of grain boundary β-phase to be analysed. High-resolution scanning electron microscopy (HR-SEM) was conducted in secondary electron (SE) mode, in order to determine the grain boundary β-phase particle radius, number density, and other information such as nearest neighbour distance and aspect ratio. To avoid any potential oxidation of fracture surfaces, imaging of specimens was conducted within 30 minutes of the Ga-embrittled fracture. Analytical image analysis was executed via the combination of Fovea Pro^TM^ and Image J^TM^ software.

A select number of sensitised samples were also examined with transmission electron microscopy (TEM), in order to supplement the HR-SEM results from fracture surfaces. The samples used for TEM were prepared by twin jet electro-polishing of 3mm discs in 30 vol. % nitric acid −70 vol.% methanol solution at −30 °C (using a Struers Tenupol 5). TEM examination was carried out using an FEI Tecnai G2 T20 (for conventional bright field images using an internal CCD camera) and FEI Tecnai F20 (for scanning TEM) both operating at 200 kV.
